# Focus on carbon-neutral energy science and technology

**DOI:** 10.1080/14686996.2018.1476219

**Published:** 2018-06-18

**Authors:** Tatsumi Ishihara, Petros Sofronis

**Affiliations:** a International Institute for Carbon-Neutral Energy Research (WPI-I^2^CNER), Kyushu University, Fukuoka, Japan

This focus issue showcases a sample of current research accomplishments at the International Institute for Carbon Neutral Energy Research (I^2^CNER) and reviews some recent developments in the area of materials for carbon-neutral energy technologies. I^2^CNER was inaugurated as the sixth institute of the World Premier International Research Center (WPI) Initiative by the Japanese Ministry for Education, Culture, Sports, Science and Technology in 2010. I^2^CNER is an international collaboration between Japan and the US, based at Kyushu University and the University of Illinois at Urbana-Champaign, respectively. At I^2^CNER, our mission is to contribute to the creation of a sustainable and environmentally friendly society by conducting fundamental research underlying current technologies that advance increased efficiency in energy conversion and energy use and reduce the carbon emission of fuel and electricity generation.

This collection of papers explores fundamental science problems in solid oxide fuel cells and electrolyzers; energy storage; fuel generation; production, storage, and utilization of hydrogen as a fuel; and CO_2_ capture membranes. We would like to draw attention to the fact that the research outlined in these papers in most cases involves researchers from multiple universities. More specifically, these collaborative efforts truly bear the mark of I^2^CNER because they were enabled by the framework of the I^2^CNER-WPI project.

In the area of oxide cells, Staykov and his collaborators seek the origin of surface electrons that participate in oxygen reduction reactions for SrO-terminated surface of SrTiO_3_ and iron-doped SrTiO_3_; Staykov et al. compared bulk density of states (DOS) to near-surface DOS. Their calculations demonstrate that the conduction band, which is formed mainly by the *d*-states of Ti, and Fe-induced states within the band gap of SrTiO_3_ are accessible only on TiO_2_-terminated SrTiO_3_ surface, while the SrO-terminated surface introduces a tunneling barrier for the electrons populating the conductance band [1]. Perry and her collaborators compare approaches to measure oxygen surface exchange kinetics, by simultaneous optical transmission relaxation (OTR) and AC-impedance spectroscopy (AC-IS) in the same mixed conducting SrTi_0.65_Fe_0.35_O_3−x_ film by accounting for the presence and condition of the metal current collectors (Pt, Au). The results suggest that, while the current collector might influence measurements by AC-IS, the OTR method offers a continuous, *in situ*, and contact-free method to measure oxygen exchange kinetics at the native surfaces of thin films [2]. The team of Kilner uses secondary ion mass spectrometry (SIMS) depth profiling to study ^18^O and ^2^H diffusion at 300 °C temperature in the double perovskite material PrBaCo_2_O_5+δ_ (PBCO) in flowing air containing 200 mbar of ^2^H_2_^16^O. The team found that PBCO still retains significant oxygen diffusivity and that the presence of water (^2^H_2_^16^O) enhances the surface exchange rate over that in pure oxygen by a factor of ~3. In addition, studying the three-dimensional ^2^H distribution, they found it to be inhomogeneous and probably related to the presence of hydrated layers in the interior walls of pores, thus not being due to proton diffusion. Kilner et al. concluded that PBCO acts mainly as an oxygen ion mixed conductor when used in proton-conducting ceramic fuel cells [3].

In the area of photelectrochemical water splitting, Ghuman explores alternative routes for the development of inexpensive and abundant catalysts for water splitting. She studied pristine and Fe^2+^-doped amorphous titanium dioxide (aTiO_2_) photocatalysts through density functional theory with Hubbard energy correction and found that pristine amorphous (a)TiO_2_ surface is capable of anchoring H_2_O molecules more strongly than the doped aTiO_2_ as well as the rutile (110) surface. In addition, she found that the water dissociation barrier on pristine aTiO_2_ catalyst is reduced significantly when aTiO_2_ is doped with Fe^2+^ [4]. Continuing her work on the chemistry of small molecules, Ogo et al. mechanistically investigated the catalytic H_2_ evolution from formic acid in water using a formate-bridged dinuclear Ru complex as a formate hydrogen lyase model [5]. Watanabe reviews the fundamental principles and recent developments in the area of hydrogen production by artificial photosynthesis. In particular, he focuses on the photocatalytic water splitting through composite organic/inorganic photocatalysts [6]. Kearney et al. review and discuss how density functional theory and finite-element device simulations can be used to gain insights into charge transport across modified photoelectrodes in photoelectrochemical water-splitting [7].

In the area of energy storage and fuel generation, Yamauchi and her co-workers propose a promising new carbon-neutral approach to energy storage (fuel generation) and power generation based on the glycolic acid (GC)/oxalic acid (OX) redox couple. Studying the catalytic activity of various transition metals, they identified Rh, Pd, Ir, and Pt to be most effective for the electrochemical oxidation of GC. In addition, Yamauchi et al. found that OX electroreduction for the selective formation of GC, which proceeds through successive two-electron reductions, is facilitated by TiO_2_ catalysts with large specific area [8]. Edalati et al. demonstrate that high density of lattice defects produced by severe plastic deformation through high-pressure torsion (HPT) underlies the development of new nanostructured hydrogen storage materials with enhanced hydrogenation kinetics and activity. Some major findings on the impact of HPT process on the hydrogen storage performance of various titanium-based and magnesium-based materials are reviewed [9].

In the area of CO_2_ capture, Taniguchi et al. investigating the development of increased permeability membranes for CO_2_ capture found that monoethanolamine (MEA) hydroxyl group is crucial for high CO_2_ separation performance. MEA can be readily immobilized in poly(vinyl alcohol) (PVA) matrix by solvent casting of aqueous mixture of PVA and amine. The CO_2_ separation performance of such MEA containing polymeric membrane with 80 wt% amine fraction (604 barrer with CO_2_ selectivity of 58.5 over H_2_) was found to be much higher than that of conventional poly(amidoamine) (PAMAM) membrane (83.7 barrer and 51.8 selectivity) under the same operation conditions of 40 °C and under 80% relative humidity. Such MEA membranes can be freestanding with thickness larger than 3 microns [10]. Selyanchyn and Fujikawa review the current state of CO_2_ separation membranes, especially from the viewpoint of thinning the selective layers and the membrane itself in order to enhance the gas flux. In particular, future directions in the study of the relation between the gas permeation and the membrane thinning are discussed [11].

In summary, this focus issue demonstrates I^2^CNER’s various developments and accomplishments at the international level. We face international challenges that should be assessed through bringing together international talent and collaboration—which is the epitome of the I^2^CNER-WPI project. We, as a community, are enthusiastic about these scientific accomplishments that can enable technology. Though there are many challenges ahead of us, we believe the WPI community is ideally positioned to identify solutions and make disruptive advances along the path toward a carbon-neutral energy society.

Tatsumi Ishihara *International Institute for Carbon-Neutral Energy Research (WPI-I2CNER), Kyushu University, Fukuoka, Japan*Petros Sofronis *International Institute for Carbon-Neutral Energy Research (WPI-I2CNER), Kyushu University, Fukuoka, Japan*
